# Ubiquitination of the ubiquitin-binding machinery: how early ESCRT components are controlled

**DOI:** 10.1042/EBC20210042

**Published:** 2022-08-05

**Authors:** Barbara Korbei

**Affiliations:** University of Natural Resources and Life Sciences, Vienna, Austria

**Keywords:** Endosomal Sorting Complexes Required for Transport, ubiquitin, ubiquitin binding domains

## Abstract

To be able to quickly and accurately respond to the environment, cells need to tightly control the amount and localization of plasma membrane proteins. The post-translation modification by the protein modifier ubiquitin is the key signal for guiding membrane-associated cargo to the lysosome/vacuole for their degradation. The machinery responsible for such sorting contains several subunits that function as ubiquitin receptors, many of which are themselves subjected to ubiquitination. This review will focus on what is currently known about the modulation of the machinery itself by ubiquitination and how this might affect its function with a special emphasis on current findings from the plant field.

## Introduction

**Figure 1:**
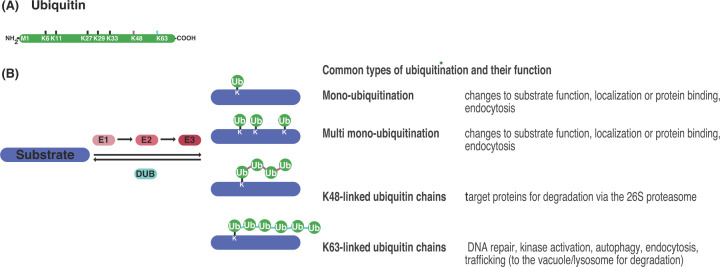
Enzymatic machinery involved in assembly and disassembly of ubiquitin chains and different ubiquitin chain topologies and their function (**A**) Ubiquitin (Ub; green circle) is a highly conserved 76-amino acid protein, where the amino-terminal methionine (M1) and seven internal lysine (K) residues (K6, K11, K27, K29, K33, K48 and K63) can serve as acceptor sites for the formation of ubiquitin chains. (**B**) In the ubiquitination process, ubiquitin is covalently conjugated to K residues of substrate proteins through the coordinated activity of ubiquitin activating (E1), ubiquitin-conjugating (E2) and ubiquitin-ligating enzyme (E3). Deubiquitinating enzymes (DUBs) remove ubiquitin from modified proteins or disassemble ubiquitin chains. Besides modification by single ubiquitin moieties (mono-ubiquitination), additional ubiquitin molecules can be ligated to a K residue of a target protein (multiple mono-ubiquitination) or a K residue of ubiquitin to form ubiquitin chains. Here the two most prominent types of chains linkage (magenta; K48, light blue; K63) are shown including a summary of the major cellular processes where specific ubiquitin linkages play a role.

In multicellular organisms, the transmission, integration and responses to environmental signals strongly depend on a tight control of plasma membrane protein function. As a response to stimuli as well as to attenuate signaling, membrane proteins are ubiquitinated and removed by a concerted process involving internalization of the proteins by endocytosis, followed by sequestration either for recycling or delivery for degradation in the vacuole/lysosome [1–4]. Substrates can be mono-ubiquitinated, which can also occur at multiple sites, or poly-ubiquitinated with a ubiquitin chain. Ubiquitin chains form via linkage of one ubiquitin to the N-terminus or internal lysine residues (lysine (K)6, 11, 27, 29, 33, 48 and 63) of another ubiquitin moiety (Figure 1A) [5,6]. Of the different types of ubiquitination that exist, mono-ubiquitination together with K63-linked poly-ubiquitin chains are thought to be crucial for targeting membrane proteins for their degradation (Figure 1B) [7–10]. Recognition of the ubiquitin moieties of cargo membrane proteins and mediation of subsequent sorting events is driven by the action of the Endosomal Sorting Complexes Required for Transport (ESCRT). This multisubunit machinery promotes extensive membrane remodeling at endosomal surfaces causing membrane invagination and scission, ultimately generating cargo-containing intralumenal vesicles (ILVs) in multivesicular bodies (MVBs) [11,12]. The ESCRT machinery consists of discrete protein complexes, with elements of the ESCRT-0, ESCRT-I and ESCRT-II recognizing and clustering ubiquitinated cargos via their ubiquitin-binding domains (UBDs), directing them to endomembrane sites, where membrane bending to form vesicles is initiated [13,14]. Target of Myb1 (TOM1) and TOM1-like (TOL) proteins are ancestral ESCRT protein families, which function instead of, or together with the ESCRT-0 to decode ubiquitin signals particularly in lineages without ESCRT-0, such as plants [15–17]. ESCRT-0, as well as the TOM1/TOLs, are the first complexes to function in the capture and concentration of ubiquitinated cargo.They recruit the ESCRT-I, which in turn binds to and recruits the ESCRT-II. The ESCRT-II directs assembly and activity of the ESCRT-III [18,19]. The ESCRT-III in turn forms heterogeneous spiral filaments to deform the membrane and is thus essential for membrane scission to produce ILVs [20,21]. ESCRT-III subunits do not interact with ubiquitin but constrict the neck of the nascent vesicle, which also serves to prevent diffusion of the deubiquitinated cargo. An AAA-ATPase and its regulators facilitate vesicle release and recycle the ESCRT-III back into its monomeric form [22,23].

Several of the ESCRT components have been found to function in processes apart from endocytic sorting [24,25] like exocytosis [26] or membrane repair and autophagy [27]. This review will focus on what is currently known about the modulation of the ESCRT machinery itself at a post-translational level by ubiquitination, which affects the function of the complex. A special focus will be placed on the early ESCRT complexes with a particular emphasis on mechanisms characterized in plants.

## Ubiquitination of UBD-containing proteins

Early-acting ESCRT complexes (ESCRT-0/TOM1/TOLs, ESCRT-I and ESCRT-II) comprise subunits that act as ubiquitin receptors, as they contain one or more UBD, which they utilize to cluster the ubiquitinated cargo and pass it on to further ESCRT complexes [4,28,29]. UBDs are heterogeneous in their organization, with modular domains of up to 150 amino acids. They are able to utilize diverse subdomains for association with ubiquitin or distinct ubiquitin chains, with their repertoire still expanding [30,31]. Typically, these domains only show low affinity towards ubiquitin [32], allowing them to rapidly and reversibly hand over cargo from one subunit to the next. Binding and specificity of such cargo recognition is enhanced by oligomerization of ubiquitin receptors, creating a high affinity and avidity cluster, as was shown for the ESCRT-0 complex [33]. Furthermore, ubiquitin receptors themselves are frequently subjected to ubiquitination in a process that depends on their UBDs and is thus referred to as coupled ubiquitination [29,34]. It can occur via interaction of the UBD with an already ubiquitinated E3 ligase, or via a ubiquitin-like domain found within certain E3 ligases or alternatively via interactions with an E2-conjugating enzyme, but mechanisms involved here remain obscure [5].

**Figure 2:**
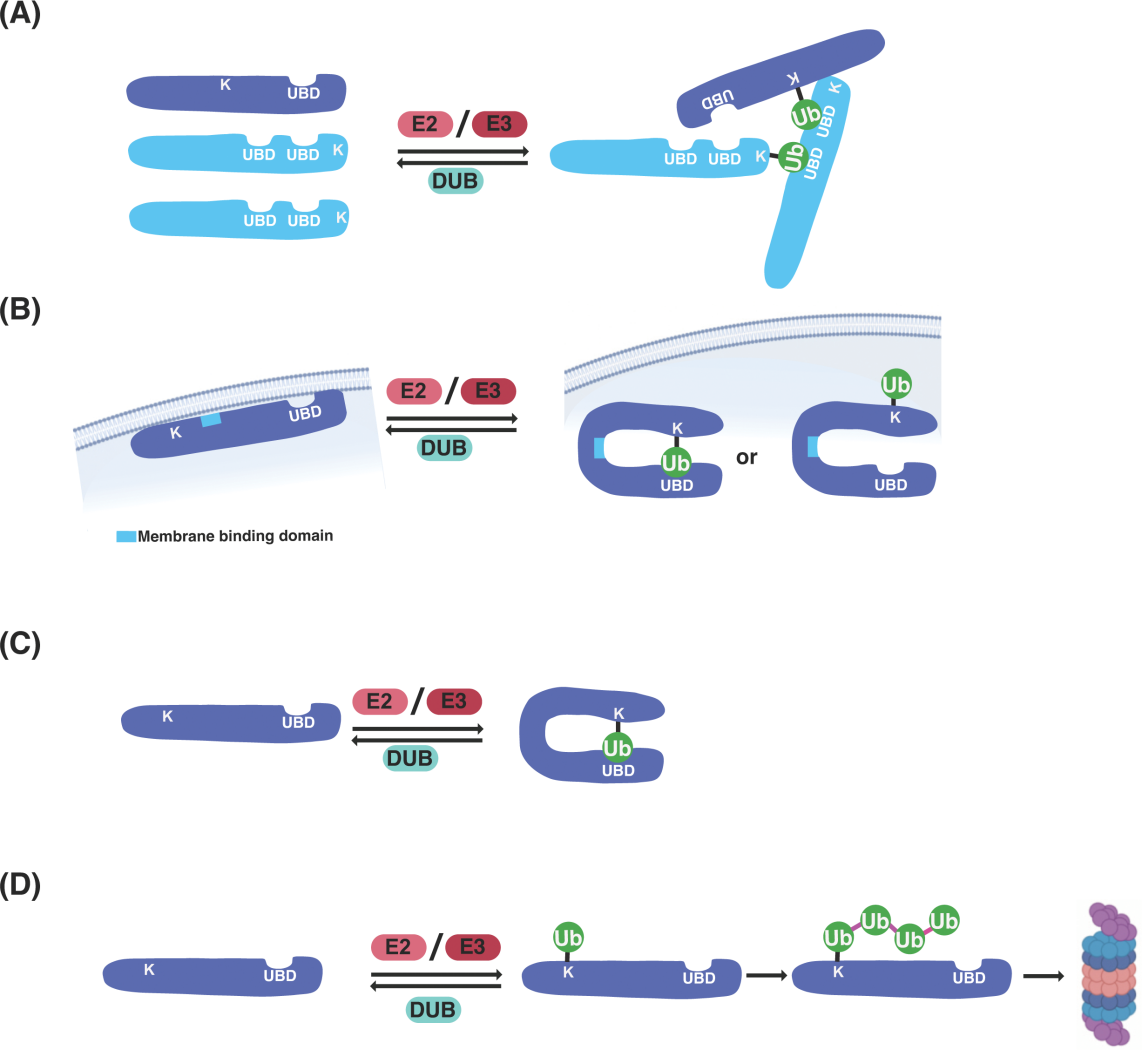
Functional implications of interactions between ubiquitin and UBD-containing protein (**A**)Protein complex assembly enabling protein oligomerization through combinatorial use of ubiquitin binding and ubiquitination. (**B**) Regulation of activity of the UBD-containing protein (exemplified by membrane binding) through conformational changes caused by ubiquitination. (**C**) Autoinhibition of the ubiquitin-binding ability of the ubiquitin receptors through self-binding. (**D**) Ubiquitination of ubiquitin receptors resulting in down-regulation by degradation.

Functional implications for this coupled ubiquitination can be diverse from affecting the activity of individual proteins to the assembly of entire complexes (Figure 2). In agreement with such diverse roles, it has been demonstrated that UBD domain proteins of the ESCRT machinery function not only in the recognition and trapping of the ubiquitinated cargo, but also in promoting and coordinating the assembly of the machinery [35]. Reversible assembly of multiprotein complexes could also be achieved through intra- and intermolecular interactions activated by ubiquitination of ESCRT machinery subunits (Figure 2A). Disassembly can consequently occur by deubiquitination through deubiquitinating enzymes (DUBs). In baker’s yeast for example, regulation of endocytic machinery assembly and disassembly, is achieved through such ubiquitination and deubiquitination of the machinery itself [36,37].

On the other hand, ubiquitination of ubiquitin receptors could function as regulatory switch, with different roles attributed to the ubiquitinated and the deubiquitinated state of such receptors (Figure 2B). The yeast endocytic ubiquitin receptor epsin interacts with its ubiquitinated cargo at the plasma membrane followed by trafficking to early endosomes. There, epsin is ubiquitinated proximal to its membrane-binding domain, which removes epsin from the endosomal membrane rendering it unable to bind its cargo. Thus, deubiquitination of epsin appears to reset its function, permitting further cycles of cargo binding and sorting [38,39]. Additionally, UBD-containing proteins, such as the ESCRT-0 subunit hepatocyte growth factor-regulated tyrosine kinase substrate (Hrs), lose their ubiquitin-binding ability once mono-ubiquitinated. This imposes an autoinhibitory conformation, rendering them unable to bind in *trans* to ubiquitinated targets, detaching them from their cargo (Figure 2C) [40]. If deubiquitinated, ubiquitin receptors would once again regain their ubiquitin-binding ability, giving this process a cyclic mode of action. Furthermore, modification of ubiquitin receptors by ubiquitination can also result in their down-regulation by degradation (Figure 2D), destabilizing the complex to which they pertain [41]. Thus, recruitment and action of E3 ligases and DUBs to ubiquitin receptors reassures association/disassociation of the UBD-dependent protein complexes or of the UBD to its cargo, constituting the cyclic nature of the activity (Figure 2) [28,42].

## Ubiquitination of the ESCRT-0/TOM1/TOLs machinery

The ESCRT-0 is the furthest upstream component of the ESCRT machinery and acts at a branchpoint in endosomal trafficking between degradation and recycling. In yeast/mammals it is made up of two subunits, the vacuolar protein sorting-associated protein 27 (Vps27)/Hrs and the Hse1 (Hbp STAM, EAST 1)/signal transducing adaptor molecule (STAM), respectively [43]. Each subunit of this complex contains several UBDs, which act cooperatively conferring avidity and specificity to the binding and commitment of the ubiquitinated cargo to the lysosomal/vacuolar degradation pathway [33].

ESCRT-0 ubiquitin receptors are ubiquitinated and the major E3-ligases involved are the HECT-type Ubiquitin ligases Nedd4 in mammals, and their yeast homologs Rsp5 [44–47]. Remarkably, although the yeast ESCRT-0 component Vps27 has been demonstrated to be ubiquitinated *in vivo*, the mechanistic significance of this modification remains vague, as removing ubiquitination by various means did not impact on the functionality of the ESCRT complex or affect the sorting process to the MVBs [44]. The mammalian version of VPS27, namely Hrs, is also ubiquitinated by E3 ligases such as Nedd4 and AIP4, a Nedd4-like E3 ubiquitin ligase [47,48]. Specifically, Hrs is mono-ubiquitinated in a process that is dependent on its UBD [49]. This coupled ubiquitination results in association of the Hrs UBD with its internal ubiquitin, rendering the protein unable to bind and thus sort ubiquitinated cargo towards the lysosome [40]. It is possible that flotillin-1 is required for the opening of this autoinhibited Hrs conformation on endosomal membranes [50].

Hse1, the other yeast subunit of the ESCRT-0 complex, associates with E3 ligase Rsp5 as well as DUBs counteracting the ubiquitination. One study claims that association with Rsp5 ubiquitinates both Vps27 and Hse1, but this is not assumed to have a regulatory function for the ESCRT-0 subunits, but rather seems involved in the regulating cargo ubiquitination [45]. In another study though, Hse1 was found to be decorated by K63-linked poly-ubiquitin chains through Rps5 activity, and this antagonizes the high-affinity interaction between K63-linked poly-ubiquitinated cargos and the UBD of Hse 1. Furthermore, this ubiquitination of Hse1 is antagonized by K63-linked ubiquitin chain-specific DUBs, resetting the ubiquitin receptor for further use [46]. STAM, the mammalian Hse1 counterpart, is ubiquitinated *in vivo* in a UBD-dependent manner presumably by Nedd4 [47]. Furthermore, STAM is responsible for interaction with endosome-associated DUBs to modulate the ubiquitination status of either cargo and/or the machinery [51]. DUBs participating, involve the K63-linked ubiquitin chain-selective DUB AMSH and the non-selective DUB USP8, which compete for binding to STAM via a shared binding site. This association is furthermore vital for activation of the DUBs [42,52,53]. In the absence of AMSH, both STAM and Hrs are strongly ubiquitinated, potentially by K63-linked ubiquitin chains, and AMSH seems responsible for regulating the activity of the ESCRT-0 proteins [54]. USP8, on the other hand, deubiquitinates and therefore protects Hrs and STAM from proteasomal degradation, maintaining the integrity of the ESCRT-0 [52,55,56].

As there are no ESCRT-0 homologs in plants, they need to be functionally substituted by members of the ancestral TOL protein family, consisting of nine proteins termed TOL1-9 in *Arabidopsis thaliana* [15,16]. TOLs bind ubiquitin, via their tandemly arranged N-terminal UBD with a preference towards K63-linked ubiquitin chains [16]. The predominantly plasma membrane-localized TOL6, was found to be ubiquitinated mainly in the soluble protein fraction *in planta*. This could be indicative of a regulatory function, as constitutive ubiquitination of TOL6 does not result in its destabilization but rather serves in relocating the protein from the plasma membrane to the cytosol, where it seems no longer functional in cargo sorting [16]. Regulation by relocation might also explain the spatiotemporal variations of TOL6 localization upon certain environmental stimuli, presumably involved in localized fine-tuning of plasma membrane protein sorting in response to such signals [15,57]. However, whether or not ubiquitination of the TOLs serves as such a regulatory switch, essential for TOL association with the ubiquitinated cargo at the plasma membrane still needs to be determined. Alternatively, TOL ubiquitination might function in its removal from a putative higher order TOL complex, similar to the ESCRT-0.

## Ubiquitination in the ESCRT-I

The ESCRT-I in yeast/mammals comprises a heterotetramer of Vp23/tumor susceptibility gene 101 (TSG101), Vps28/VPS28, Vps37/VPS37 and multivesicular body sorting factor 12 (Mvb12)/MVB12 and its ortholog ubiquitin-associated protein 1 (UBAP1), of which only Vps23/TSG101 and Mvb12/MVB12/UBAP1 contain known UBDs. In yeast, little is known about the ubiquitination of ESCRT-I subunits. Vps23 is targeted by Rsp5 and deubiquitinated by a DUB with a specificity for K63-linked ubiquitin chains [46]. Vps36, the only UBD-containing ESCRT-II subunit, which recruits the ESCRT-II to the ESCRT-I via this UBD [18] may also be modified by K63-linked ubiquitin chains through Rsp5 [46].

For the mammalian TSG101, a tight control of its amount and localization has been demonstrated to be crucial for the function of this essential ESCRT-I component, where depletion or overproduction can cause disruption of vital processes [58]. TSG101 levels are regulated post-translationally by ubiquitination mediated by at least three different E3 ubiquitin ligases: mouse double minute 2 homolog (MDM2), TSG101-associated ligase (TAL), and Mahogunin Ring Finger-1 (MGRN1). MDM2 ubiquitinates TSG101 and targets it for proteasomal degradation [59]. TAL binds via its tandem tetrapeptide motif (PTAP) to the ubiquitin-conjugating enzyme E2 variant (UEV) domain of TSG101. One report stated that TAL mono-ubiquitinates TSG101 at multiple unidentified lysine residues, causing it to relocate from a membrane-bound active form to an inactive soluble form [60]. Furthermore, if lysines in the C-terminus of TSG101 are accessible, TAL might catalyze its poly-ubiquitination, resulting in the proteasomal degradation of TSG101 [41]. Availability of these lysines is prevented by complex formation of TSG101 with additional subunits of the ESCRT-I like VPS37 or VPS28. This process ensures that excess TSG101, not incorporated into the ESCRT-I complex is degraded, thus enforcing a stringent control of protein homeostasis [41]. The E3 ubiquitin ligase MGRN1 possesses a single conserved PSAP sequence that can bind to the UEV domain of TSG101 [61]. MGRN1 mono-ubiquitinates TSG101 on multiple sites, which may not have a significant role in controlling the level of the TSG101 protein, but rather modifies trafficking and functionality of TSG101 [61], which might also concern its function in autophagy [62].

In plants, the ESCRT-I complex is similar to that of yeast/mammals, with the exception that no Mvb12-like proteins have been found. Thus, VPS23 is the only UBD-containing ESCRT-I protein, with two isoforms VPS23A and B identified in plant genomes [2,4]. VPS23A is decorated by K48-linked poly-ubiquitin chains and degraded via the 26S proteasome pathway. The UEV domain of VPS23A physically interacts via two PSAP motifs with the E3 ligase XB3 ORTHOLOG 5 in *Arabidopsis thaliana* (XBAT35). XBAT35 functions in ABA signaling, where VPS23A is epistatic to XBAT35 [63]. Ubiquitination of a plant-specific ESCRT component, which contains a UBD and interacts with ESCRT-I and III subunits, called FYVE1/FREE1 (FYVE DOMAIN PROTEIN REQUIRED FOR ENDOSOMAL SORTING 1) has been shown [64]. In response to iron deficiency, the E3 ubiquitin ligases, SINA OF *Arabidopsis thaliana* (SINAT) interacts with and ubiquitinates FYVE1/FREE1 promoting its degradation, while by contrast, a SINAT protein family member lacking ubiquitin ligase activity, protects FYVE1/FREE1 from ubiquitination and stabilizes it [64]. Thus ESCRT-I subunits in plants also seem to undergo dynamic ubiquitination particularly in response to environmental stimuli.

## Conclusion and outlook

The sequestration of plasma membrane proteins into the degradation pathway needs to be both precise and stringent to prevent promiscuous protein degradation or incorrect recycling of proteins destined to be degraded. Although stringency of binding is required, it also needs to be reversible, so the cargo can be passed on within the machinery. This can be achieved by multimerizing different UBD-containing subunits of the machinery, providing flexibility and avidity in ubiquitin chain recognition by the ESCRT pathway as seen for the ESCRT-0 complex [65]. In plants, whether the TOL proteins, alone or potentially in combination with other UBD proteins are subjected to a similar oligomerization is currently not known. The plant-specific UBD protein SH3P2 has been found to bind K63-linked ubiquitin chains of cargo in proximity to the plasma membranes as well as ESCRT-I subunits. It furthermore interacts with the DUB AMSH3, which plays a role in membrane protein degradation in plants [66,67]. Ubiquitination of SH3P2 or whether if functions together with the TOLs has not been shown to date. Furthermore, the archetypical endocytic receptors proteins in plants do not contain known UBDs [68–70], thus also the build-up of the endocytic machinery in plants still needs closer scrutiny, with potential novel UBDs to be discovered or other ubiquitin receptors substituting for them [30].

Although the idea that UBD proteins are themselves subjected to ubiquitination, which may either act in degradation or affect protein functionality, has been around for two decades [49], a precise assessment of why and when UBD proteins are ubiquitinated has not been obtained. The association of many different E3 ligases and DUBs with the ESCRT machinery, with a functionality besides their role in cargo ubiquitination or deubiquitination, indicates that the regulation of UBD proteins through ubiquitination is of crucial importance. Nevertheless, especially in single-cell organisms like yeast, preventing ubiquitination of ESCRT-0 and ESCRT-I subunits does not block their ability to transport ubiquitinated cargo to the vacuole/lysosome [44]. Thus, potentially the additional regulation of ESCRT subunits by ubiquitination to subtly affect their role in cargo degradation could be a way of fine-tuning the response of a cell to its environment in higher order organisms.

Future research to elucidate the role of ubiquitination of UBD-containing proteins will be of exceptional interest especially with respect to understanding how higher organisms integrate environmental responses into cellular outputs by regulating the abundance of plasma membrane proteins.

## Summary

- Reversible ubiquitination of ubiquitin receptors presumably serves to regulate the needs to be stringent and versatile, but also reversible.
- A higher order organization of the ESCRT machinery, in which ubiquitin receptors clearly play an essential role, is vital especially for the correct function of the ESCRT-0 machinery. The role UBD proteins play in complex assembly and if a similar higher order complexes exist in plants, still needs to be determined.
- How changes in the environment are translated into fine-tuning of the ESCRT machinery potentially by affection ubiquitination of UBD-containing proteins will be exciting research topics for the future.

## Abbreviations

DUB: deubiquitinating enzyme
ESCRT: endosomal sorting complexes required for transport
Hrs: hepatocyte growth factor-regulated tyrosine kinase substrate
ILV: intralumenal vesicle
MDM2: mouse double minute 2 homolog
MGRN1: Mahogunin Ring Finger-1
MVB: multivesicular body
Mvb12: multivesicular body sorting factor 12
STAM: signal transducing adaptor molecule
TAL: TSG101-associated ligase
TOL: TOM1-like
TOM1: Target of Myb1
TSG101: tumor susceptibility gene 101
UBD: ubiquitin-binding domain
UEV: ubiquitin-conjugating enzyme E2 variant
Vps27: vacuolar protein sorting-associated protein 27
